# Cost-effectiveness analysis of pharmaceutical care in adult critically ill patients: based on a prospective cohort study

**DOI:** 10.3389/fphar.2024.1446834

**Published:** 2024-07-19

**Authors:** Chunyan Wei, Ming Hu, Guanghui Chen, Zhijing Yan, Wanhong Yin, Zhiang Wu

**Affiliations:** ^1^ West China School of Pharmacy, Sichuan University, Chengdu, China; ^2^ Department of Pharmacy, West China Hospital, Sichuan University, Chengdu, China; ^3^ Department of Critical Care Medicine, West China Hospital, Sichuan University, Chengdu, China; ^4^ West China School of Clinical Medical College, Sichuan University, Chengdu, China; ^5^ Yihong College of Business, Shenyang Pharmaceutical University, Peking, China

**Keywords:** pharmaceutical care, critically ill, health outcome, economic, cost-effectiveness analysis

## Abstract

**Background:**

The medication regimen for critically ill patients is complex and dynamic, leading to a high incidence of drug-related problems. This study aimed to assess the effectiveness and economic efficiency of pharmaceutical care for these patients.

**Methods:**

In this prospective cohort study conducted in a tertiary hospital, adult patients were assigned either to a clinical pharmaceutical care group or a control group based on existing clinical grouping rules. Health outcomes and economic indicators were collected, followed by a cost-effectiveness analysis.

**Results:**

The acceptance rate for clinical pharmacist interventions was 89.31%. The pharmaceutical care group exhibited significant reductions in the rate of medication errors (40.65% vs. 61.69%, *P* < 0.001) and adverse drug events (44.52% vs. 56.45%, *P* = 0.020). The usage rates for special-grade antibiotics (85.16% vs. 91.13%, *P* = 0.009) and proton pump inhibitors (77.42% vs. 88.71%, *P* = 0.002) were also lower in the pharmaceutical care group. Secondary outcomes did not show significant differences in total hospital stay (21 days vs. 22 days, *P* = 0.092). However, ICU stay was significantly shorter (9 days vs. 11 days, *P* = 0.003) in the pharmaceutical care group. Cost-effectiveness analysis demonstrated that each 1% reduction in adverse drug events associated with ICU pharmaceutical care saved $226.75 in ICU hospitalization costs and $203.42 in total ICU drug costs. A 1% reduction in the medication error rate saved $128.57 in ICU hospitalization costs and $115.34 in total ICU drug costs.

**Conclusions:**

Pharmaceutical care significantly reduces adverse drug events and medication errors, promotes rational use of medications, decreases the length of ICU stay, and lowers treatment costs in critically ill patients, establishing a definitive advantage in terms of cost-effectiveness.

## 1 Introduction

Treatment options for patients in intensive care units (ICUs) are complex, and drug-related problems (DRPs) are frequent occurrences. The incidence of medication errors (MEs) and adverse drug events (ADEs) in ICUs is significantly higher than in non-ICU wards (Otero-López et al., 2006). A systematic review demonstrated that the incidence of MEs in ICUs varied from 8.1 to 2,344 per 1,000 patient days, while ADEs ranged from 5.1 to 87.5 per 1,000 patient days (Wilmer et al., 2010). Additionally, ICU treatment incurs significant medical expenses. In 2010, ICU care represented 13.2% of U.S. hospital costs and 4.1% of national health expenditures. The cost of critical care rose from 0.54% to 0.72% of the gross domestic product (GDP), an increase of $4.7 trillion, and was expected to continue to rise (Halpern et al., 2016). An Institute of Medicine report titled “To Err is Human: Building a Safer Health System” highlighted those accidental errors led to 98,000 deaths annually, with MEs contributing to 19%, resulting in approximately 7,000 deaths (Brennan et al., 1991; Phillips et al., 1998; Kruer et al., 2014). A recent report indicated that the U.S. spends more than $40 billion annually on patients affected by MEs and more than $21 billion on preventable MEs (Silva et al., 2016). Rothschild et al. (2005) found that 45% of adverse events could be prevented, noting that the most serious adverse events often occurred during drug use, primarily due to negligence and mistakes rather than inadequate knowledge. China data are limited; however, single-center observational studies have indicated that the incidence rate of MEs was 124.7 per 1,000 patient days.

Pharmaceutical care includes pharmacists’ professional services, including medication education and recommendations for adjusting treatment regimens based on patients’ specific conditions. This approach aims to enhance the efficacy and safety of drug therapy (van Mil et al., 2013; Mishore et al., 2020). In 2000, the Society of Critical Care Medicine and the American College of Clinical Pharmacy defined the role of clinical pharmacists in the ICU (Rudis et al., 2000). This role includes identifying medication histories, evaluating medications, monitoring pharmacokinetics, assessing indications for parenteral nutrition, providing educational training to other ICU staff and residents, monitoring investigational drug use, monitoring ADEs, and serving on medication boards. Previous studies have indicated that pharmaceutical care can reduce the incidence of DRPs and mortality in ICU patients. Furthermore, an increased patient-to-pharmacist ratio, indicating more patients per clinician, is significantly associated with a longer ICU stay (LOS) (Toukhy et al., 2021; Sikora et al., 2022).

Clinical pharmacists represent a distinct group of hospital pharmacists dedicated to promoting the rational use of drugs. In China, these professionals must complete 1 year of professional training to qualify. The importance of pharmaceutical care is increasingly recognized within the healthcare system. In 2018, China’s National Health Commission mandated a change in pharmaceutical care from a “drug-centered” approach to a “patient-centered” approach. Specific studies in various areas of the disease have shown that implementing pharmaceutical care strategies, such as medication therapy management (MTM), improves both patient outcomes and economic efficiency (Bukhsh et al., 2018; Salman et al., 2021). However, research primarily focuses on patients with chronic diseases, with limited studies addressing critically ill patients (Chamorro-de-Vega et al., 2020; Abousheishaa et al., 2022). This oversight may be due to the complex conditions and various prognostic factors associated with ICU patients (Cheng et al., 2019; Lang et al., 2020). Over the past three decades, the role of clinical pharmacists in ICUs has evolved, and although their contributions are significant, their specific functions often remain undefined.

In 2024, China will officially implement a pharmaceutical care payment policy, where patients will directly pay for pharmacist services. Consequently, whether pharmaceutical care leads to an increase or reduction in treatment costs is a topic worthy of discussion. This prospective cohort study aimed to describe the scope of pharmaceutical care in the ICU, assess its effectiveness and economic impact, and provide evidence to support the value of pharmaceutical care in this setting.

## 2 Methods

### 2.1 Research design

This prospective cohort study was conducted in a tertiary hospital in China. Due to the limited number of clinical pharmacists, participation in all medical units was impossible. Clinical pharmacists participated in rounds within specific medical units. The unit with clinical pharmacist participation was designated as the pharmaceutical care group (intervention group), and those without participation were designated as control groups. Three medical units were selected, each with the same level of ICU patient admissions, identical numbers of beds, similar teams of first-line physicians, and other care configurations managed by different medical leaders. To mitigate the effects of patient heterogeneity, patients of the same type were treated in these units and randomly assigned to the units based on bed availability. According to daily operational principles, the patients were allocated to either the pharmaceutical care group with clinical pharmacist involvement (unit 1) or to conventional treatment groups without such involvement (units 2 and 3).

The research hospital’s ethics committee approved the study in June 2022 (Approval No. 2022-975). The authorized trustees of all patients admitted to the target medical units were communicated with and informed of the objectives and potential risks of the study, and informed consents were obtained from all participants.

### 2.2 Participants

Patients admitted to the ICU from October 2022 to April 2023 were included in the study. The inclusion criterion was admission to the ICU for any reason. Exclusion criteria included: 1) an ICU stay of less than 3 days, considering that patients with stays shorter than 3 days were extremely critical and died within 3 days of admission or their condition was mild and they were primarily postoperative patients, 2) being under the age of 18 years, and 3) pregnancy.

### 2.3 Intervention and control program

Pharmaceutical Care Group (Intervention Group): Two clinical pharmacists participated in the evaluation of therapeutic drugs and attended rounding at the patient’s wards. One was a certified clinical pharmacist who participated in rounds three times a week, and the other was a training clinical pharmacist who participated twice a week. A training clinical pharmacist is defined as a pharmacist who possesses a professional background in pharmacy but has not yet obtained the requisite certification. This individual is required to perform duties under the supervision of a certified clinical pharmacist. The certified clinical pharmacist approved the intervention suggestions made by the training clinical pharmacist. Clinical pharmacists assessed patients’ conditions and treatment regimens during ward rounds, making treatment suggestions. Specific procedures include 1) assistance in drug selection and dosage adjustments, particularly for antibiotics; 2) evaluation of indications for and duration of antibiotic and proton pump inhibitor (PPI) therapies; 3) evaluation of dosages of sedative and analgesic drugs; 4) reminding physicians to perform therapeutic drug monitoring (TDM); 5) evaluation of potential ADEs; and 6) intervention in MEs. Clinical pharmacists conducted detailed medication evaluations, prepared comprehensive medication adjustment recommendations, and discussed these with the medical team during rounds. The guidelines most frequently referred to for pharmaceutical interventions are listed in Supplementary Table S1.

Control Group: Usual care was administered. In both the pharmaceutical care group and the control group, basic pharmaceutical care, such as prescription dispensing, reviews of prescriptions through the electronic system, and pharmaceutical consultations, was performed as required by medical institutions. However, physicians primarily completed the formulation of treatment plans, adjustment of drug doses, evaluation of adverse drug reactions, monitoring of therapeutic drug concentrations, and identification of MEs.

### 2.4 Sample size calculation

The following formula was used to calculate the sample size. In the formula, $Z_{\alpha }$ is the bilateral Z value corresponding to the test level α. In this study, α = 0.05, and the corresponding $Z_{\alpha }$ is 1.96. $Z_{\beta }$ is the unilateral Z value corresponding to the β value. β = 0.10, and the corresponding $Z_{\beta }$ is 1.28. $p_{1}$ is the therapeutic effectiveness rate of the pharmaceutical care group. $p_{2}$ is the therapeutic effectiveness rate of the control group. $\bar{p}$ represents the overall therapeutic efficacy rate. According to Sun Li’s research (Sun, 2019), $p_{1}$ is 83.02%, $p_{2}$ is 66.38%, and $\bar{p}$ is 74.53%. The resulting sample size was 142.

$$n={\left(\frac{Z_{\alpha }\sqrt{2\left(1-\bar{p}\right)\bar{p}}+Z_{\beta }\sqrt{p_{1}\left(1-p_{1}\right)+p_{2}\left(1-p_{2}\right)}}{p_{1}-p_{2}}\right)}^{2}$$

### 2.5 Data collection

Patients’ basic information, intervention process indicators, health outcomes, and economic indicators were collected systematically. Intervention process indicators mainly describe the steps involved in the intervention. Given the multitude of factors influencing the clinical prognosis of critically ill patients, such as extracorporeal life support, rehabilitation therapy, nutritional support, and respiratory support therapy, pharmaceutical care was focused on drug-related aspects. Due to the non-randomized nature of this study, caution must be exercised when interpreting the effects of pharmaceutical care on patient mortality and length of hospital stay. Consequently, the rate of therapeutic effectiveness, length of stay, and mortality were designated secondary outcomes, while indicators directly related to pharmacist intervention were classified as primary outcomes. These included the appropriate use of antibiotics and PPIs, TDM, adverse drug reactions, and MEs observed during pharmacist interventions.

Economic evaluations were conducted from the perspective of medical institutions, incorporating direct medical costs and pharmacist labor costs. Details of health outcomes and economic indicators are provided in Table 1. To mitigate selective reporting, researchers responsible for data collection did not participate in pharmaceutical care. Conversion from Chinese Yuan (CNY) to dollars was calculated at 7.24.

**TABLE 1:** Classification and indicators of the collected data.

Classification	Indicators
Baseline information	• Sex
	• Age
	• Weight
	• APACHE II score^a^
	• Mechanical ventilation rate
	• First diagnosis in the ICU
Intervention process indicators	• Number of interventions by pharmacists
	• Intervention acceptance rate by physicians
Health outcomes	Primary outcomes
	• Antibiotic use (utilization rate of antibiotics, utilization rate of special-grade antibiotics, course of antibiotic use)
	• PPI use (PPI utilization rate, PPI treatment course, new upper gastrointestinal bleeding)
	• Therapeutic drug concentration monitoring (therapeutic drug concentration monitoring, drug concentration up to the standard rate)
	• Frequency and incidence of adverse drug events
	• Medication error^b^ (medication error rate, number of medication errors, days of medication errors, days *per capita* of medication errors)
	Secondary outcomes
	• Therapeutic effective rate
	• Length of stay (total length of stay, length of ICU stay)
	• Course of mechanical ventilation
	• Mortality
Economic indicators	• Hospitalization cost (total hospitalization cost, ICU hospitalization cost)
	• Drug cost (total drug cost, ICU drug cost)
	• Average daily labor cost of a pharmacist
	• ICER of the above health outcome indicators

^a^ APACHE II score: a classification system for disease severity and predicting patient mortality.

^b^ The rule for calculating the medication error rate is that patients with at least one medication error for all the medications used are classified into the medication error group.

Patients transferred out of the ICU due to improvement were considered to have received effective treatment. Diagnosing adverse drug reactions was challenging. When patients exhibited abnormal test results and clinical signs, disease factors were first ruled out before evaluating potential drug causes. If drugs were suspected, the incidence of adverse reactions was recorded. In the intervention group, physicians and pharmacists conducted adverse reaction assessments jointly, while in the control group, they were exclusively completed by physicians.

According to the China National Health Commission, special-grade antibiotics are broad-spectrum and potent antibiotics that are costly, more likely to cause adverse drug reactions, and can induce bacterial resistance. These antibiotics require careful monitoring and management, with the specific agents used listed in Supplementary Table S2. The duration of antibiotic use was counted only during ICU admissions. The PPIs included were omeprazole, esomeprazole, pantoprazole, and ilaprazole.

### 2.6 Statistical analysis

SPSS 26.0 was used for statistical analysis. Quantitative indicators are calculated as the mean value and standard deviation, while categorical indicators are presented with the number and percentage. The normally distributed quantitative data were analyzed using the t-test, whereas non-normally distributed data were assessed using nonparametric tests. Categorical data were evaluated using the chi-square test. All statistical tests were conducted bilaterally, unless otherwise specified. A *p*-value of 0.05 or less was considered to indicate statistical significance.

### 2.7 Cost-effectiveness analysis

The incidence rate of ADEs and the ME rate were assessed as health outcomes. Economic indicators were represented by ICU hospitalization costs and total ICU drug costs. The incremental cost-effectiveness ratio (ICER) was calculated to analyze cost-effectiveness, as outlined by Cazarim et al. (2018). The formula to calculate the ICER is dividing the incremental ICU hospitalization costs (or incremental total ICU drug costs) by the rate of improvement in MEs and ADEs. Given that the study period did not exceed 1 year, costs were not discounted.

## 3 Results

### 3.1 Basic patient information

The study included 155 patients in the pharmaceutical care group and 248 patients in the control group, satisfying the requirement of a statistical sample size (Table 2). No significant differences were observed in sex or body weight between the groups, with a higher proportion of men than women in both groups. The average age of the individuals in the control group was slightly higher than that of the pharmaceutical care group (57.46 ± 17.38 years vs. 53.42 ± 17.95 years, *P* = 0.025). However, there were no significant differences in APACHE II scores between the groups (18.14 ± 7.89 vs. 18.66 ± 8.40, *P* = 0.533). The rates of mechanical ventilation and the primary diagnoses upon ICU admission did not differ significantly between the groups.

**TABLE 2:** The basic information of patients.

Basic information	Pharmaceutical care group	Control group	*P*
Sample size	155	248	—
Sex (male/female)	112/43	172/76	0.534
Age (years)	53.42 ± 17.95	57.46 ± 17.38	0.025
Body weight (kg)	67.53 ± 17.20	65.16 ± 14.32	0.369
APACHE II score	18.66 ± 8.40	18.14 ± 7.89	0.533
Mechanical ventilation rate (%)	98.71	95.16	0.058
First diagnosis in the ICU			
• Sepsis/septic shock	102	158	0.669
• Severe trauma/empyrosis	22	32	0.711
• Malignant tumor	13	28	0.348
• Other systemic diseases	18	30	0.884

### 3.2 Summary of pharmacist intervention

In the pharmaceutical care group, clinical pharmacists performed 393 interventions on 119 patients, achieving an intervention coverage rate of 76.77% (the number of patients receiving interventions divided by the total number of patients). Of these, 351 interventions were accepted, resulting in an intervention acceptance rate of 89.31%. Pharmacist interventions included seven main aspects, detailed in Table 3. The most frequent interventions related to the route of administration and dosage, comprising 52.16% (205/393) of all interventions. The next most common were interventions concerning the duration of the medication, which accounted for 22.14% (87/393) of the interventions. The acceptance rate for most types of interventions exceeded 80%. However, interventions related to medication indications had the lowest acceptance rate, at 63.64%.

**TABLE 3:** Summary of interventions.

Intervention type	Number of interventions (N)	Number of interventions received (n)	Intervention acceptance rate (%)
Route of administration and dosage	205	187	91.22
Duration of medication	87	76	87.36
TDM^a^	30	28	93.33
Adverse drug events evaluation	24	24	100.00
Medication indication	22	14	63.64
Drug selection	20	17	85.00
Inappropriate combination of drugs	5	5	100.00
Total	393	351	89.31

^a^ Therapeutic drug monitoring.

### 3.3 Influence of pharmacist intervention on health outcomes

As detailed in Table 4, concerning primary outcomes, the antibiotic utilization rate did not show differences between the two groups, with only one patient in the control group not treated with antibiotics. However, the utilization rate of special-grade antibiotics was significantly lower in the pharmaceutical care group compared to the control group (85.16% vs. 91.13%, *P* = 0.009). The duration of antibiotic treatment averaged 11.71 ± 8.29 days in the pharmaceutical care group and 14.90 ± 12.65 days in the control group, although this difference was not statistically significant. The reduction in the duration of antibiotic treatment was primarily attributed to shorter stays in the ICU.

**TABLE 4:** Comparison of health outcomes.

Types of indicators		Pharmaceutical care group	Control group	*P*
Primary outcomes				
Antibiotic use	Utilization rate of antibiotics (n/%)	155/100.00	247/99.60	0.429
	Utilization rate of special-grade antibiotics (n/%)	132/85.16	226/91.13	0.009
	Course of antibiotic therapy (days)	11.71 ± 8.29	14.90 ± 12.65	0.053
PPI use	PPI utilization rate (n/%)	120/77.42	220/88.71	0.002
	PPI treatment course (days)	6.15 ± 6.31	8.57 ± 8.48	0.003
	New upper gastrointestinal bleeding	3	18	0.019
TDM	TDM monitoring rate (n/%)	86/55.48	121/48.79	0.191
	Rate of TDM reaching the standard (n/%)	100/67.11	241/60.86	0.179
ADEs	Incidence of adverse drug reactions (n/%)	69/44.52	140/56.45	0.020
	Number of adverse drug reactions	86	202	0.038
MEs	Medication error rate (n/%)	63/40.65	153/61.69	<0.001
	Number of medication errors	92	249	<0.001
	Accumulated days of medication errors (days)	208	1,173	<0.001
	Number of days *per capita* of medication errors (days)	1.34	4.73	<0.001
Secondary outcomes				
Total length of stay (median, days)		21	22	0.092
Length of ICU stay (median, days)		9	11	0.003
Therapeutic effective rate (%)		93/60.00	154/62.10	0.674
Course of mechanical ventilation (median, days)		7	9	0.118
Mortality (%)		21/13.55	40/16.13	0.482

For PPIs, both the utilization rate (77.42% vs. 88.71%, *P* = 0.002) and the duration of treatment (6.15 ± 6.31 days vs. 8.57 ± 8.48 days, *P* = 0.003) were significantly lower in the pharmaceutical care group. The incidence of gastrointestinal bleeding events was significantly reduced in the pharmaceutical care group compared to the control group (3 vs. 18, *P* = 0.019).

In terms of TDM, the monitoring rate (55.48% vs. 48.79%, *P* = 0.191) and the compliance rate (67.11% vs. 60.86%, *P* = 0.179) were higher in the pharmaceutical care group, although the differences were not statistically significant.

ADEs were significantly lower in the pharmaceutical care group both in terms of incidence (44.52% vs. 56.42%, *P* = 0.020) and number (86 cases vs. 202 cases, *P* = 0.038). Pharmacist care significantly reduced both the rate of MEs (40.65% vs. 61.69%, *P* < 0.001) and the duration of MEs (208 days vs. 1,173 days, *P* < 0.001), with the number of days per capita with MEs decreasing from 4.73 days to 1.34 days (*P* < 0.001).

Secondary outcome statistical analysis indicated that the length of ICU stay was significantly shorter in the pharmaceutical care group (9 days vs. 11 days, *P* = 0.003). While the total hospital stay was also shorter in the pharmaceutical care group compared to the control group, this difference was not statistically significant (21 days vs. 22 days, *P* = 0.092). Other outcomes, including mechanical ventilation duration, treatment response rate, and mortality rate, did not show significant differences between the groups.

### 3.4 Influence of pharmacist intervention on economic indicators

The labor cost for the pharmacists was calculated by multiplying the average daily income of the two clinical pharmacists by their average working time, which was estimated at 5 h per day. This time included both medication evaluation prior to ward rounds and participation during rounds. As a result, the daily labor cost in the pharmaceutical care group was $34.67, while in the control group it was $5.66. Detailed calculations are provided in Table 5.

**TABLE 5:** Calculation method for the pharmacist labor cost.

Classification	Professional pharmacist	Training pharmacist
Pharmaceutical care group		
Average daily wage ($)	75.97	24.86
Labor time (hours)	5	5
Working days per week (days)	3	2
Daily labor cost^a^ ($)	34.67	
Control group		
Average daily wage ($)	75.97	24.86
Labor time (hours)	1	1
Working days per week (days)	2	3
Daily labor cost^b^ ($)	5.66	

^a^ Average daily labor cost of pharmacists in the pharmaceutical care group = average daily wage of professional pharmacist × 5/8 (5 of the 8 working hours per day are devoted to pharmaceutical intervention) × 3/5 (3 out of 5 days a week participating in pharmaceutical interventions) + average daily wage of training pharmacist × 5/8 (5 of the 8 working hours per day are devoted to pharmaceutical intervention) × 2/5 (2 out of 5 days a week participating in pharmaceutical interventions).

^b^ Average daily labor cost of pharmacists in the control group = average daily wage of professional pharmacist × 1/8 (1 of the 8 working hours per day are devoted to pharmaceutical interventions) × 2/5 (participation in pharmaceutical interventions 2 out of 5 days a week) + average daily wage of training pharmacist × 1/8 (1 of the 8 working hours per day are devoted to pharmaceutical intervention) × 3/5 (participation in pharmaceutical interventions 3 out of 5 days a week).

Regarding drug costs, the total in the pharmaceutical care group was significantly lower than in the control group ($16,303.08 ± $15,657.63 vs. $20,391.16 ± $19,141.25, *P* = 0.027). The total hospitalization cost was also lower in the pharmaceutical care group compared to the control group, although this difference was not statistically significant ($35,819.58 ± $28,814.26 vs. $41,965.81 ± $33,651.61, *P* = 0.060). ICU hospitalization costs ($20,997.28 ± $19,616.34 vs. $23,702.39 ± $22,924.90, *P* = 0.337) and ICU drug costs ($12,984.22 ± $13,785.40 vs. $15,738.87 ± $16,483.34, *P* = 0.079) in the pharmaceutical care group were also lower than in the control group, although these differences were not statistically significant. The average labor cost for pharmacists in the pharmaceutical care group was $415.35 ± $290.31 (Table 6).

**TABLE 6:** Economic indicator comparison (the unit is $).

Types of indicators	Pharmaceutical care group ($)	Control group ($)	*P*
Total hospitalization cost	35,819.58 ± 28,814.26	41,965.81 ± 33,651.61	0.060
Total drug cost	16,303.08 ± 15,657.63	20,391.16 ± 19,141.25	0.027
ICU hospitalization cost	20,997.28 ± 19,616.34	23,702.39 ± 22,924.90	0.337
Average ICU drug cost	12,984.22 ± 13,785.40	15,738.87 ± 16,483.34	0.079
Average pharmacist labor cost	415.35 ± 290.31	87.46 ± 73.02	<0.001
Total ICU drug cost^a^	13,399.57 ± 14,008.83	15,826.33 ± 16,543.37	0.157

^a^ Total ICU drug cost = average ICU drug cost + average pharmacist labor cost.

### 3.5 Cost-effectiveness analysis

Statistical analysis indicated that ICU pharmaceutical care positively impacted almost all health outcomes, although not all differences reached statistical significance. The ICU hospitalization cost, average ICU drug cost, and total ICU drug cost were consistently lower in the pharmaceutical care group, indicating the absolute economic advantage of pharmaceutical care. To assess the value of pharmaceutical care, we calculated the incremental cost associated with the incidence of adverse drug reactions and the medication error rate in the pharmaceutical care group compared to the control group. The results of this cost-effectiveness analysis are presented in Table 7. During the implementation of pharmaceutical care in the ICU, each 1% reduction in ADEs resulted in savings of $226.75 in ICU hospitalization costs and $203.42 in total ICU drug costs. Similarly, a 1% reduction in MEs corresponded to savings of $128.57 in ICU hospitalization costs and $115.34 in total ICU drug costs.

**TABLE 7:** Cost-effectiveness analysis results.

Indicators	Outcomes		
	Pharmaceutical care group	Control group	Increment
Incidence of ADEs	44.52	56.45	11.93
Incidence of MEs	40.65	61.69	21.04
ICU hospitalization cost ($)	20,997.28	23,702.39	−2705.11
Total ICU drug cost	13,399.57	15,826.33	−2426.76
ICER_1_ ^a^ ($/%)	−226.75		
ICER_2_ ^b^ ($/%)	−203.42		
ICER_3_ ^c^ ($/%)	−128.57		
ICER_4_ ^d^ ($/%)	−115.34		

^a^ ICER_1_ = Incremental ICU hospitalization costs/rate of improvement in adverse drug events.

^b^ ICER_2_ = Incremental total ICU drug costs/rate of improvement in adverse drug events.

^c^ ICER_3_ = Incremental ICU hospitalization costs/rate of improvement in medication errors.

^d^ ICER_4_ = Incremental total ICU drug costs/rate of improvement in medication errors.

## 4 Discussion

In 2024, the World Health Organization (WHO) introduced the “Patient Safety Rights Charter,” emphasizing that more than 50% of injuries in healthcare settings could be prevented (World Health Organization, 2024). Given the specific needs of ICU patients, ensuring their medication safety is a critical concern. The European Society of Critical Care Medicine highlighted the need for pharmacy professionals in the ICU through an article published in Intensive Care Medicine, citing ten reasons, including the promotion of rational drug use and the benefits of pharmacovigilance (McKenzie et al., 2024). Although the economic value of ICU pharmaceutical care is widely acknowledged, comprehensive economic evaluations remain scarce, with most studies focusing solely on cost-benefit analyses and lacking cost-effectiveness assessments (Bosma et al., 2018; Crosby et al., 2023). Our research addresses this gap by demonstrating improvements in both health outcomes and economic indicators in the pharmaceutical care group compared to the control group.

Antibiotics are crucial in ICU settings, and their rational use is essential to improve treatment efficacy and mitigate bacterial resistance (Timsit et al., 2019). Our results indicate that while the pharmacist intervention did not decrease the rate or duration of antibiotic use, it effectively reduced the use of special-grade antibiotics. These antibiotics, reserved for use by specially qualified physicians in China, play a significant role in combating antimicrobial resistance (Mancuso, 2021; Dambroso-Altafini, 2022). Despite this, overuse of antibiotics in the ICU remains a significant issue, with empirical therapy often not adequately adjusted even in confirmed cases of sepsis (Timsit, 2019).

The misuse of PPIs, which are now under national surveillance in China, is a known problem globally, with their overuse linked to an increased incidence of hospital-acquired pneumonia in ICU settings (Wang et al., 2020; Dharmarajan, 2021). Our findings suggest that clinical pharmacist involvement significantly reduced both the treatment duration and utilization rate of PPIs, as well as the incidence of new upper gastrointestinal tract bleeding. This improvement is attributed to the pharmacists’ rational evaluation of medication instructions, course, and dosage, providing more effective treatment recommendations.

These results demonstrate the valuable role of pharmaceutical care in improving the rational use of PPIs. However, the high usage rate of PPIs persists, highlighting an ongoing challenge in ICU settings. There is an urgent need for guidelines aimed at promoting the rational use of PPIs among ICU patients to further refine treatment protocols and optimize patient outcomes.

In the pharmaceutical care group, the incidence of MEs and ADEs was significantly lower than in the control group, which could reduce treatment costs and improve therapeutic outcomes. Despite this improvement, the incidence of MEs and ADEs in the ICU remained high, exceeding 40%, reflecting the complex conditions of critically ill patients, the diversity of therapeutic drugs used, and the variable treatment plans. This demonstrates the need for more robust methods to mitigate MEs and ADEs, such as improving electronic medical records systems. Due to the implementation of an electronic pre-prescription review system, minimal significant drug-drug interactions were observed, occurring four times in the pharmaceutical care group and five times in the control group. The most frequent interactions involved the combination of carbapenem and valproic acid (6 cases), followed by caspofungin and dexamethasone without dose adjustment (2 cases), and a single occurrence of the imipenem and ganciclovir interaction.

Total hospitalization and ICU costs were lower in the pharmaceutical care group. Cost-effectiveness analysis indicated that ICU pharmaceutical care not only reduces the incidence of ADEs and MEs but also provides substantial economic benefits. Given these findings, the extension of medical insurance coverage to include pharmaceutical care costs is advocated to promote a wider adoption of ICU pharmaceutical care.

However, some outcomes, such as the length of ICU stay, did not show significant differences, which may be attributed to variances in medical decision-making between units. For example, different medical leaders might vary in their approach to tracheal intubation removal and patient discharge from the ICU. Additionally, reductions in ICU stay could be linked to fewer MEs and ADEs, making drug treatments more effective and safer.

This study has several limitations. First, to minimize disruption to routine clinical practices, a non-randomized, real-world grouping approach was utilized. All patients in the target medical units from October 2022 to April 2023 who met the inclusion criteria were included, showing no significant baseline differences in APACHE II scores, mechanical ventilation rates, or initial ICU diagnoses. Although a significant age difference was observed, the prognostic significance of the APACHE II score is considered to outweigh that of age. Second, the calculation of pharmacist labor costs was based on the average earnings of the two participating pharmacists, limiting the generalizability of the findings. Lastly, the study did not categorize included diseases. Considering the varying prognoses associated with different diseases, disease categorization could enhance the study’s referential value. Future research should focus on conducting more rigorous randomized controlled trials to further validate the benefits of pharmaceutical care for critically ill patients. A broader investigation into the labor costs of Chinese pharmacists in different medical institutions is recommended to provide a more representative benchmark.

## 5 Conclusion

The involvement of pharmacists in the care of critically ill patients has been shown to reduce the incidence of ADEs and MEs, as well as enhance the management of special-grade antibiotics and PPIs. Pharmaceutical care in the ICU demonstrates substantial cost-effectiveness. Nevertheless, the methodologies employed in follow-up research require further refinement. Additional strategies must be implemented to prevent MEs and ADEs among critically ill patients.

## Data Availability

The raw data supporting the conclusions of this article will be made available by the authors, without undue reservation.
