# Supplementary material for: Cost-effectiveness analysis of pharmaceutical care in adult critically ill patients: based on a prospective cohort study
Source: Front Pharmacol. 2024 Jul 19;15:1446834. doi: 10.3389/fphar.2024.1446834 (PMC11294147; doi:10.3389/fphar.2024.1446834)
Supplement: Supplementary file 1 [file Table1.docx]

Supplementary Material

## Supplementary Table 1

**Supplemental Table 1**. Reference guidelines for pharmaceutical intervention

| **NO.** | **Publisher** | **Publication time (year)** | **Name** |
| --- | --- | --- | --- |
| 1 | Chinese College of Emergency Physicians | 2018 | Guidelines for emergency treatment of sepsis/septic shock in China |
| 2 | Society of Critical Care Medicine | 2016 | Surviving Sepsis Campaign: International Guidelines for Management of Sepsis and Septic Shock |
| 3 | National Health Commission of the People's Republic of China | 2020 | Guidelines for clinical application of proton pump inhibitors |
| 4 | Infectious Diseases Society of America | 2022 | Guidance on the Treatment of Antimicrobial-Resistant Gram-Negative Infections |
| 5 | Spanish Society of Infectious Diseases and Clinical Microbiology and Infectious Diseases | 2022 | Executive summary of the consensus document of the Spanish Society of Infectious Diseases and Clinical Microbiology (SEIMC) on the diagnosis and antimicrobial treatment of infections due to carbapenem-resistant Gram-negative bacteria |
| 6 | American College of Gastroenterology | 2021 | ACG Clinical Guideline: Upper Gastrointestinal and Ulcer Bleeding |
| 7 | American Society of Hematology | 2020 | American Society of Hematology 2020 guidelines for management of venous thromboembolism: treatment of deep vein thrombosis and pulmonary embolism |
| 8 | Critical Care Medicine Society of Chinese Medical Association | 2018 | Guidelines for analgesia and sedation in adult ICU patients |
| 9 | Respiratory Society of Chinese Medical Association | 2016 | Guidelines for the diagnosis and treatment of community-acquired pneumonia in adults in China |
| 10 | Respiratory Society of Chinese Medical Association | 2018 | Guidelines for diagnosis and treatment of hospital-acquired pneumonia and ventilator-associated pneumonia in adults in China |
| 11 | Surgery Society of Chinese Medical Association | 2019 | Guidelines for Diagnosis and treatment of abdominal infection in China |
| 12 | Surgery Society of Chinese Medical Association | 2021 | Chinese guidelines for Diagnosis and treatment of acute pancreatitis |

## Supplementary Table 2

**Supplemental Table 2**. Special-grade antibiotics involved in the study.

| **Number** | **Name** | **Number** | **Name** |
| --- | --- | --- | --- |
| 1 | Cefperazone-Sulbactam | 2 | Piperacillin-Sulbactam |
| 3 | Aztreonam | 4 | Ceftazidime-Avibactam |
| 5 | Imipenem-Cilastatin | 6 | Meropenem |
| 7 | Tigecycline | 8 | Colistin sulfate |
| 9 | Voriconazole | 10 | Micafungin |
| 11 | Caspofungin | 12 | Amphotericin B |
